# The Memorial Sloan Kettering Prognostic Score: Correlation with survival in patients with advanced gastric cancer

**DOI:** 10.1002/cam4.6608

**Published:** 2023-10-03

**Authors:** Mingyu Wan, Yongfeng Ding, Xiaolu Ma, Xiaoyu Chen, Xin Xu, Chenyu Mao, Jiong Qian, Cheng Xiao, Haiping Jiang, Yulong Zheng, Lisong Teng, Nong Xu

**Affiliations:** ^1^ Department of Medical Oncology The First Affiliated Hospital of Zhejiang University Hangzhou China; ^2^ Department of Surgical Oncology The First Affiliated Hospital of Zhejiang University Hangzhou China

**Keywords:** gastric cancer, memorial Sloan Kettering prognostic score, prognosis

## Abstract

**Background:**

Notwithstanding that the past decade has witnessed unprecedented medical progress, gastric cancer (GC) remains a leading cause of cancer death, highlighting the need for effective prognostic markers. The Memorial Sloan Kettering Prognostic Score (MPS) has been validated as a valuable prognostic tool for patients with metastatic pancreatic adenocarcinoma (mPDAC). This study aimed to assess the prognostic value of the MPS in advanced GC.

**Methods:**

Data from 367 patients were analyzed in the present study. The MPS for each patient was calculated based on the sum of scores based on the neutrophil‐to‐lymphocyte ratio and serum albumin levels. Multivariate analyses were performed to identify the independent clinicopathological parameters associated with overall survival (OS). Further subgroup analyses based on clinicopathological features were conducted.

**Results:**

Patients with MPS 0 (*n* = 161), MPS 1 (*n* = 158), and MPS 2 (*n* = 48) exhibited significantly different OS, with a median survival duration of 20.7 (95%CI: 12.2–29.2), 14.9 (95%CI: 12.5–17.3), and 12.7 (95%CI: 9.3–16.0) months, respectively (*p* < 0.001). Significant differences in survival were observed among different groups of patients receiving chemotherapy (18.5 months vs. 14.7 months vs. 11.0 months, *p* = 0.03) or the subgroup receiving chemotherapy plus immunotherapy as first‐line treatment (32.6 months vs. 17.7 months vs. 12.7 months, *p* = 0.02). The MPS was identified as an independent prognostic factor in multivariate analysis. During subgroup analyses, MPS‐low (MPS 0) was consistently associated with a better prognosis than MPS‐high (MPS 1 or 2).

**Conclusions:**

MPS is a practical, simple, and useful prognostic tool for patients with advanced GC. Further studies are warranted to validate its prognostic value in advanced GC.

## 1 INTRODUCTION

Gastric cancer (GC) is the fourth leading cause of cancer death worldwide, with over 769,000 documented deaths in 2020.^1^ Although the overall prognosis has improved over the past decade, the 5‐year survival rate of GC remains poor. Given the poor specificity of early symptoms, most GC patients are diagnosed at advanced stages, requiring systemic treatment. The emergency of immune checkpoint inhibitor therapy has rapidly changed the landscape of advanced GC treatment.^2^ However, the prognosis of some patients remains dismal. It is now understood that traditional prognostic markers, including CEA,^3^ AFP,^4^ and LDH,^5^ exhibit low specificity, making them suboptimal in the modern era of immunotherapy. Although prognostic factors, including PD‐L1 expression,^6^ tumor mutation burden (TMB),^7^ microsatellite instability (MSI),^8^ microRNA (miR/miRNA),^9^ and epigenetic alterations^10^ have been developed to predict prognosis in GC, biochemical markers that can effectively predict patient outcomes remain largely unknown. In addition, the widespread application of certain molecular biomarkers in clinical practice is hindered by their high cost, time‐consuming nature, and limited availability. Therefore, a cheap and easily accessible prognostic model for advanced GC patients that can predict the survival prognosis of patients is urgently required.

Various malignant tumors have been observed to originate from sites characterized by infection, chronic irritation, and inflammation. The tumor microenvironment (TME), consisting of inflammatory cells, plays a significant role in facilitating the proliferation and progression of malignant tumors.^11^, ^12^ Inflammatory cells, along with their secreted chemokines and cytokines, play a pivotal role in shaping the TME. These elements exert significant influences on the growth, migration, and differentiation of various cell populations within the TME, influencing the effectiveness of adaptive immunity and anti‐tumor therapies.^13^, ^14^ Serum albumin level serves as a vital parameter in clinical practice for evaluating the nutritional status of patients afflicted with malignant tumors. Additionally, it exhibits a close association with disease status, inflammation, and liver function.^15^ Prior studies have consistently shown a strong correlation between low serum albumin levels and poor prognostic outcomes in individuals with malignant tumors.^16^, ^17^, ^18^ From these observations, the combination of nutritional parameter and biomarker associated with inflammation may be good predictors of the survival prognosis of patients with malignant tumor.

In recent years, Lebenthal et al. introduced the Memorial Sloan Kettering Prognostic Score (MPS) as a novel scoring system to assess the prognosis of patients diagnosed with metastatic pancreatic adenocarcinoma (mPDAC), effectively stratifying patients according to their prognosis.^19^, ^20^ This prognostic scoring system assigns a score of either 0 or 1 to two variables: the serum albumin level (score 0 for an albumin level ≥4 g/dL, score 1 for an albumin level <4 g/dL) and the neutrophil‐to‐lymphocyte ratio (NLR; score 0 for an NLR ≤4, score 1 for an NLR >4). Three different groups of patients were identified, with a median survival of 12.9 (95%CI, 10.9–14.3), 9.0 (95%CI, 7.4–10.3), and 5.4 (95%CI, 4.3–6.6) months for MPS of 0, 1, and 2, respectively. Increasing evidence showed that the MPS may be effective for other tumor types. Wang et al. demonstrated that the NLR and serum albumin could be combined to provide additional risk stratification for ovarian cancer,^21^ it has also been reported that the combination of serum albumin and NLR was significantly associated with OS and disease‐free survival (DFS) of GC patients submitted to curative surgery.^22^, ^23^ However, whether MPS is a predictor of prognosis of advanced GC has not yet been investigated. Therefore, we performed a retrospective study to examine the prognostic significance of the MPS on the survival outcomes of patients with advanced GC. Our aim was to develop a simple and cost‐effective model that could assist in identifying patients who may benefit from treatment.

## 2 METHODS

### 2.1 Study design and patients

This retrospective study enrolled patients diagnosed with advanced or metastatic gastric or gastro‐esophageal junction cancer at the Medical Oncology Department of the First Affiliated Hospital of Zhejiang University between January 2010 and June 2021. The inclusion criteria were as follows: (1) histologically confirmed, locally advanced, inoperable, recurrent, or metastatic gastric adenocarcinoma; (2) received no previous systemic treatment or had disease progression (PD) more than 6 months after adjuvant therapy or postoperative adjuvant chemotherapy; (3) at least one treatment efficacy assessment. The exclusion criteria were as follows: (1) pretreatment complete blood count values not obtained 2 weeks before treatment initiation; (2) double primary cancers; (3) recent operation; (4) infectious diseases; (5) incomplete medical records and missing complete blood count data. All patients received chemotherapy with or without immune checkpoint inhibitors as first‐line treatment. The chemotherapy regimens were as follows: oxaliplatin plus capecitabine (XELOX), S‐1 plus paclitaxel (SPA), oxaliplatin plus fluorouracil and leucovorin (FOLFOX), and S‐1 plus oxaliplatin (SOX). The dosage was determined according to the actual condition and preference of the patients. The protocol was approved by the Ethics Committee of Zhejiang University (reference number: 2022–871).

### 2.2 Data collection

Pathological and clinical features of the included patients were collected. Peripheral blood was collected in the early morning within 2 weeks before the start of treatment. Data were abstracted from an electronic medical record system. Computed tomography (CT) and/or magnetic resonance imaging (MRI) were performed at baseline and every 2 or 3 cycles. Overall survival (OS) was defined as the time from initial treatment until death. The NLR was defined as the ratio between the neutrophil and lymphocyte counts. In the MPS, two variables, the serum albumin level and NLR, were assigned a prognostic score of either 0 or 1. A score of 0 was given for an albumin level ≥4 g/dL, and a score of 1 was given for an albumin level <4 g/dL. Similarly, a score of 0 was assigned for an NLR ≤4, while a score of 1 was assigned for an NLR >4. The MPS was calculated by summing the scores of these two variables.

### 2.3 Statistical analysis

The Kaplan–Meier method was used to analyze OS, and the log‐rank test was used to compare differences in OS. The median follow‐up was calculated using the reverse Kaplan–Meier method. Multivariate analysis was performed using the Cox proportional hazards model (Forward LR method), with the following variables included: sex (male vs. female), age (<60 vs. ≥60), differentiation (poor vs. moderate‐well), intra‐abdominal metastasis (no vs. yes), location (GEJ vs. Non‐GEJ), history of GC surgery (no vs. yes), type of therapy (chemotherapy vs. combination therapy), history of smoke (no vs. yes), history of alcohol (no vs. yes), hypertension (no vs. yes), diabetes (no vs. yes), and the MPS. Receiver operating characteristics (ROC) curve analysis was performed, and the area under the curve (AUC) was calculated. Subsequently, calibration plots for the MPS were generated to predict the probability of 1‐year and 2‐year survival. Statistical analyses were performed on R language (R Core Team), Stata software 17.0 (Stata Corp.), SPSS 26.0 (SPSS, Inc.), and GraphPad Prism 9.0 (GraphPad Software).

## 3 RESULTS

### 3.1 Characteristics of patients

**FIGURE 1:**
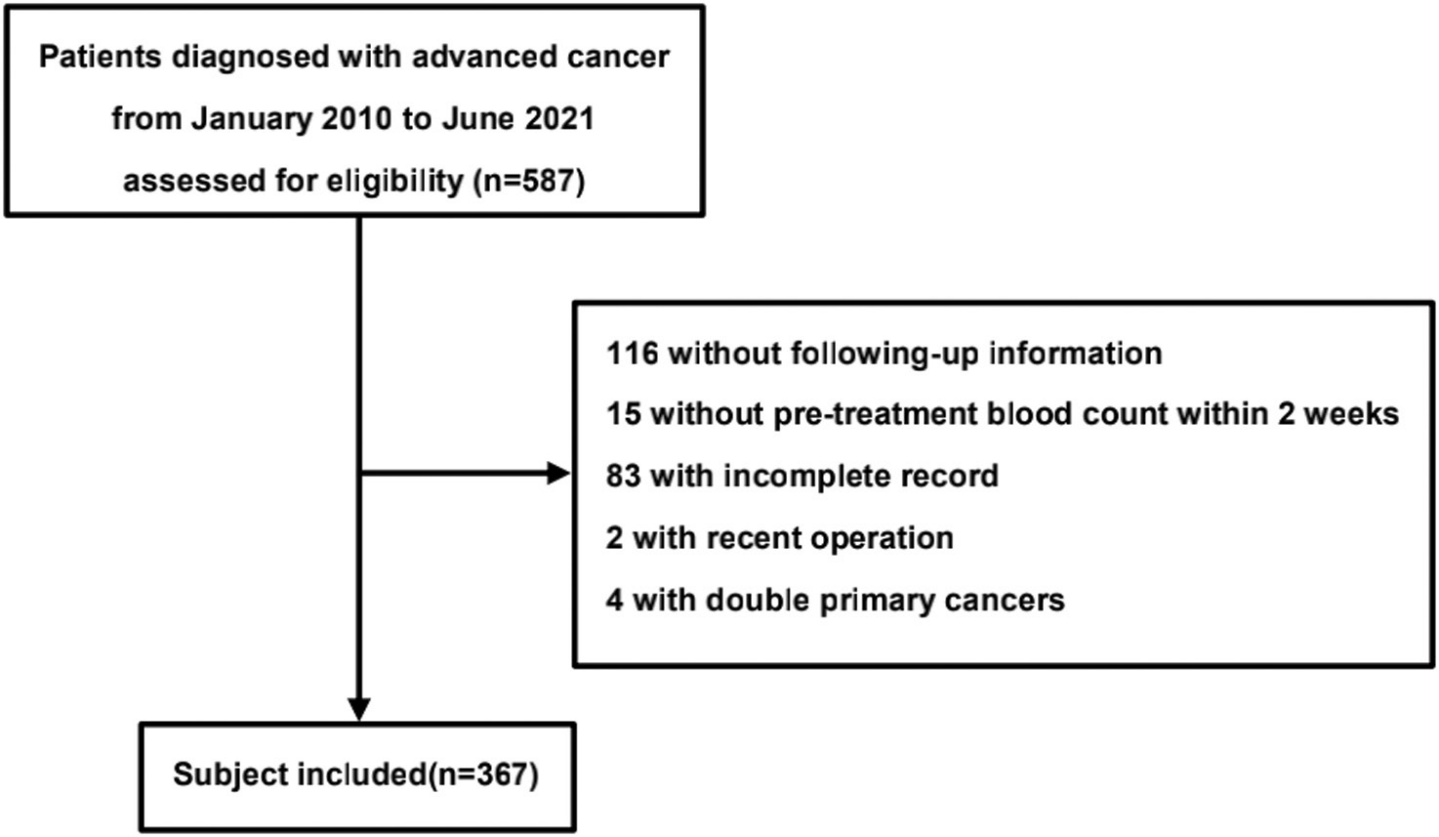
Flowchart visualizing the inclusion and exclusion of study subjects.

Figure 1 shows the flowchart outlining the patient selection process. Patients were excluded due to missing follow‐up information (*n* = 116), unavailability of pretreatment complete blood count within 2 weeks (*n* = 15), incomplete records (*n* = 83), recent operation (*n* = 2), and double primary cancers (*n* = 4). Pathological and clinical details of patients are summarized in Table 1. The median duration of treatment was 7 cycles. The median follow‐up time was 25.4(95% CI: 21.9–28.8) months, while the median OS was 17.1(95% CI: 15.1–19.2) months. Among 367 patients, the majority of patients were male (*n* = 267, 72.8%), had a history of GC surgery (*n* = 136, 37.1%) and received chemotherapy as monotherapy (267, 72.8%). Most patients had an MPS of 0 (*n* = 161, 43.9%), followed by MPS 1 (*n* = 158, 43.1%) and MPS 2 (*n* = 48, 13.1%). There were 45(12.3%) cases of gastroesophageal junction (GEJ) cancer and 170 (46.3%) with poor differentiation. The most common chemotherapy regimens used were SOX (36.5%) and SPA (33.8%). 98 (26.7%) patients were treated with a combination of anti–PD‐1 antibodies, and 2 (0.5%) patients received anti‐PD‐L1 antibodies (Table 1).

**TABLE 1:** Pathological and clinical characteristics of the patients.

Characteristic	*N* = 367
Age	
<60	169(46.0%)
≥60	198(54.0%)
Sex	
Male	267(72.8%)
Female	100(27.2%)
Differentiation	
Poor	170(46.3%)
Moderate‐well	182(49.6%)
Unknown	15(4.1%)
Location	
GEJ	45(12.3%)
Non‐GEJ	309(84.2%)
Unknown	13(3.5%)
Stage	
III	12(3.3%)
IV	355(96.7%)
Intra‐abdominal metastasis	
Yes	224(61.0%)
No	143(39.0%)
History of GC surgery	
Yes	136(37.1%)
No	231(62.9%)
History of smoke	
Yes	132(36.0%)
No	234(63.8%)
Unknown	1(0.3%)
History of alcohol	
Yes	104(28.3%)
No	262(71.4%)
Unknown	1(0.3%)
Hypertension	
Yes	70(19.1%)
No	296(80.7%)
Unknown	1(0.3%)
Diabetes	
Yes	27(7.4%)
No	339(92.4%)
Unknown	1(0.3%)
BMI	
≥18.5 kg/m^2^	253(68.9%)
<18.5 kg/m^2^	56(15.3%)
Unknown	58(15.8%)
NLR	
<4	106(28.9%)
≤4	261(71.1%)
Albumin	
≥4	219(59.7%)
<4	148(40.3%)
MPS	
0	161(43.9%)
1	158(43.1%)
2	48(13.1%)
Type of first‐line therapy	
Chemotherapy	267(72.8%)
Immunotherapy combined with chemotherapy	100(27.2%)
Chemotherapy	
SOX	134(36.5%)
SPA	124(33.8%)
FOLFOX	44(12.0%)
XELOX	64(17.4%)
Others	1(0.3%)
Immunotherapy	
Anti–PD‐L1 antibody	2(0.5%)
Anti–PD‐1 antibody	98(26.7%)

Abbreviations: FOLFOX, oxaliplatin plus fluorouracil and leucovorin; GEJ, gastroesophageal junction; GC, gastric cancer; NLR, neutrophil‐to‐lymphocyte ratio; MPS, Memorial Sloan Kettering Prognostic Score; SOX, S‐1 plus oxaliplatin, SPA, S‐1 plus paclitaxel; XELOX, oxaliplatin plus capecitabine.

### 3.2 Prognostic significance of the MPS in advanced GC

**FIGURE 2:**
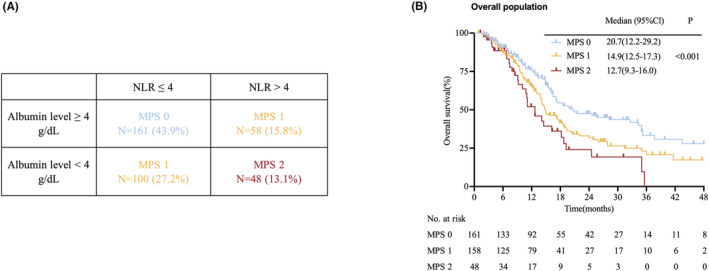
(A) Correlation analysis between the neutrophil‐to‐lymphocyte ratio (NLR) and serum albumin; (B) Kaplan–Meier curves for OS in all patients. MPS, Memorial Sloan Kettering Prognostic Score; OS, overall survival.

Patients with elevated NLR levels (>4) and reduced albumin levels (<4 g/dL) were allocated a score of 2, and those with only one of these biochemical abnormalities were allocated a score of 1. Patients with neither of these abnormalities were allocated a score of 0 (Figure 2A). Figure 2B shows the OS curve of the total 367 patients with advanced GC, according to their pretreatment MPS. The median OS was 20.7 (95%CI: 12.2–29.2), 14.9 (95%CI: 12.5–17.3), and 12.7 (95%CI: 9.3–16.0) months for MPS 0 (*n* = 161), MPS 1 (*n* = 158), and MPS 2 (*n* = 48) (*p* < 0.001). The OS rates for patients with MPS 0, 1, and 2 were 75.8%, 65.6%, and 52.0% at 1 year, while the corresponding OS rates at 2 years were 47.4%, 31.8%, and 24.1%, respectively.

**FIGURE 3:**
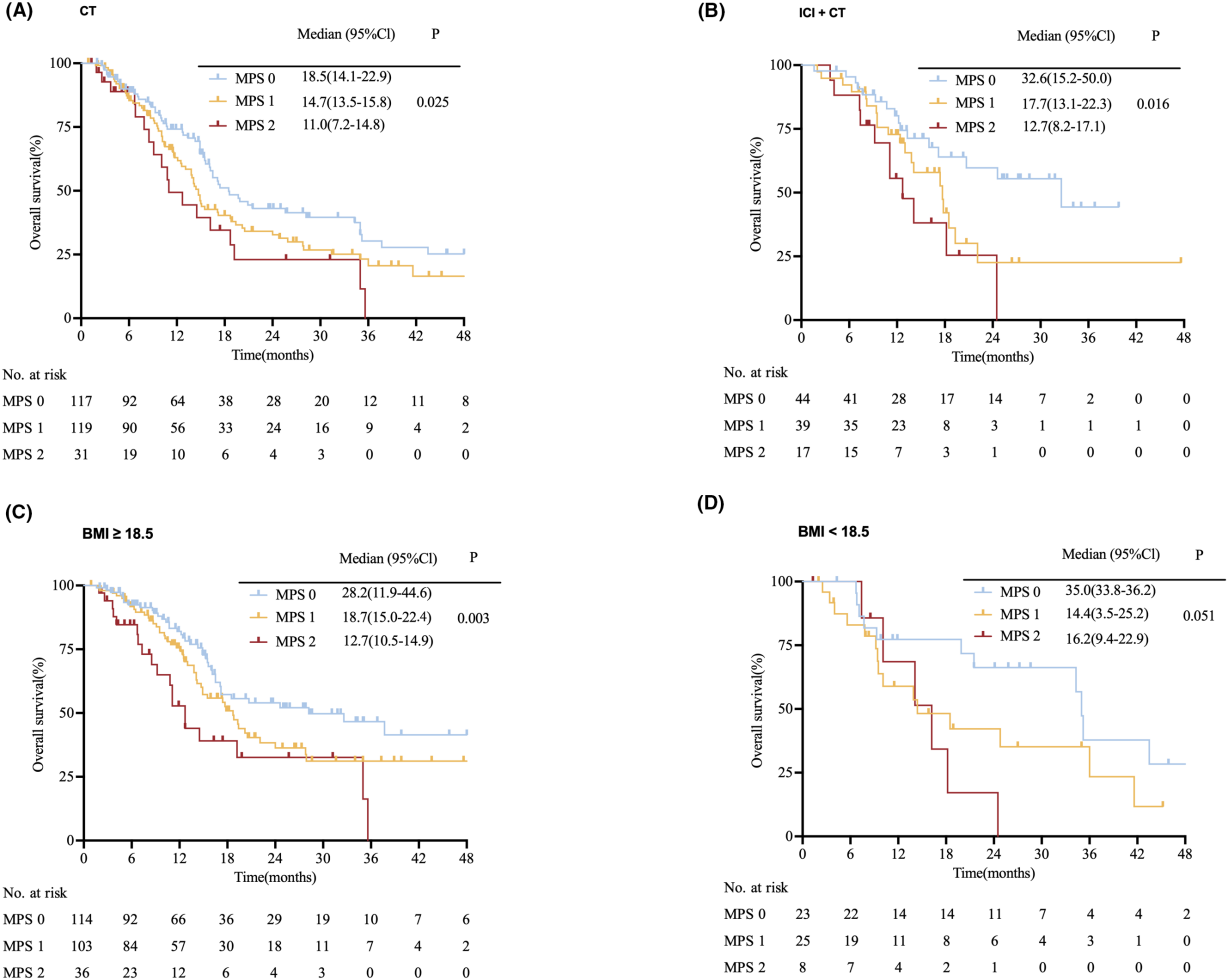
Subgroup survival analysis according to the MPS in patients with advanced GC. (A) Kaplan–Meier curves for OS in 267 patients who received chemotherapy alone; (B) Kaplan–Meier curves for OS in 100 patients who received immunotherapy plus chemotherapy; (C) Kaplan–Meier curves for OS in 253 patients with BMI ≥18.5 kg/m^2^; (D) Kaplan–Meier curves for OS in 56 patients with BMI <18.5 kg/m^2^. BMI, body mass index; MPS, Memorial Sloan Kettering Prognostic Score; OS, overall survival.

We then divided patients with advanced GC into two subgroups based on whether immune checkpoint inhibitors treatment was used as first‐line treatment. Similar trends were noted in subgroup analyses among different treatments. In the chemotherapy‐only subgroup, patients with MPS 0 (*n* = 117), 1 (*n* = 119) or 2 (*n* = 31) had significantly different prognoses, with median OS of 18.5(95%CI: 14.1–22.9), 14.7(95%CI: 13.5–15.8), and 11.0 (95%CI: 7.2–14.8) months, respectively (*p* = 0.03; Figure 3A). Among patients exposed to immune checkpoint inhibitors combined with chemotherapy, the median OS was 32.6 (95%CI: 15.2–50.0) months for MPS 0 (*n* = 44), 17.7 (95%CI: 13.1–22.3) months for MPS 1 (*n* = 39), and 12.7 (95%CI: 8.2–17.1) months for MPS 2 (*n* = 17) (*p* = 0.02; Figure 3B). In addition, patients were stratified into two groups based on baseline body mass index (BMI). In the BMI ≥18.5 kg/m^2^ subgroup, high MPS was associated with poor prognosis (*n* = 253; 28.2 months vs. 18.7 months vs. 12.7 months; *p* = 0.003; Figure 3C). In the low BMI (<18.5 kg/m^2^) subgroup, patients with MPS 0 had longer median OS than those with MPS 1 or 2, although no significant difference was observed (*n* = 56; 35.0 months vs. 14.4 months vs. 16.2 months; *p* = 0.05; Figure 3D). Additionally, we collected the PFS of these patients (*n* = 363) and observed a significantly longer PFS in patients with an MPS score of 0 compared to those with an MPS score of 1 or 2 (Figure S1). The median PFS was 8.7 (95%CI: 7.1–10.4), 7.1 (95%CI: 6.5–7.6), and 6.0 (95%CI: 4.8–7.3) months for MPS 0 (*n* = 158), MPS 1 (*n* = 157), and MPS 2 (*n* = 48) (*p* = 0.01). We also observed a similar trend in the subgroup of patients who received chemotherapy or combined therapy, although the results did not reach statistical significance (Figure S2).

### 3.3 Score validation

The results of univariate and multivariate proportional hazard analyses conducted on all 367 patients between baseline clinicopathological characteristics and survival are shown in Table 2. Univariate predictors of OS were diabetes (HR: 1.64, 95% CI: 1.02–2.64; *p* = 0.004), history of GC surgery (HR: 0.62, 95% CI: 0.46–0.83; *p* = 0.001), and the MPS (*p* = 0.001). Multivariate analysis revealed that history of GC surgery (HR: 0.66, 95% CI: 0.49–0.89; *p* = 0.007) and MPS (*p* = 0.004) were independent prognostic factors for OS. Compared with patients with MPS 0, an elevated HR was observed for patients with MPS 1 (HR: 1.46, 95% CI: 1.07–1.99) and MPS 2 (HR: 2.01, 95% CI 1.30–3.11) during multivariate analysis.

**TABLE 2:** Univariate and multivariate analysis of clinicopathologic variables in relation to OS in advanced GC patients.

	Univariate analysis				Multivariate analysis		
	*N*	HR	95%CI	*p*‐value	HR	95%CI	*p*‐value
Sex	367			0.138			
Male		Ref					
Female		1.26	0.93–1.70				
Age	367			0.495			
<60		Ref					
≥60		0.91	0.69–1.20				
Differentiation	352			0.46			
Poor		Ref					
Moderation‐Well		0.9	0.68–1.19				
Intra‐abdominal metastasis	367			0.848			
No		Ref					
Yes		1.03	0.77–1.37				
History of smoke	366			0.33			
No		Ref					
Yes		1.15	0.87–1.54				
History of alcohol	366			0.409			
No		Ref					
Yes		1.14	0.84–1.54				
Hypertension	366			0.092			0.391
No		Ref			Ref		
Yes		1.35	0.95–1.91		1.17	0.81–1.69	
Diabetes	366			0.004			0.290
No		Ref			Ref		
Yes		1.64	1.02–2.64		1.31	0.80–2.15	
Location	354			0.91			
GEJ		Ref					
Non‐GEJ		1.03	0.67–1.57				
History of GC surgery	367			0.001			0.007
0		Ref			Ref		
1		0.62	0.46–0.83		0.66	0.49–0.89	
MPS	367			0.001			0.004
0		Ref			Ref		
1		1.50	1.10–2.03		1.46	1.07–1.99	
2		2.14	1.39–3.29		2.01	1.30–3.11	

Abbreviations: GC, gastric cancer; GEJ, gastroesophageal junction; MPS, Memorial Sloan Kettering Prognostic Score.

The AUC of the MPS for predicting 1‐year, 2‐year, and 3‐year OS was 0.592, 0.607, and 0.638, respectively (Figure S3). Calibration plots depicting the probability for 1‐year (A) and 2‐year (B) survival are presented in Figure S2, whereby the diagonal 45° dashed line represents ideal prediction performance, the solid line indicates the performance of the MPS and vertical bars indicate 95% confidence intervals (Figure S4).

### 3.4 The MPS predicts outcomes in different subgroups

**FIGURE 4:**
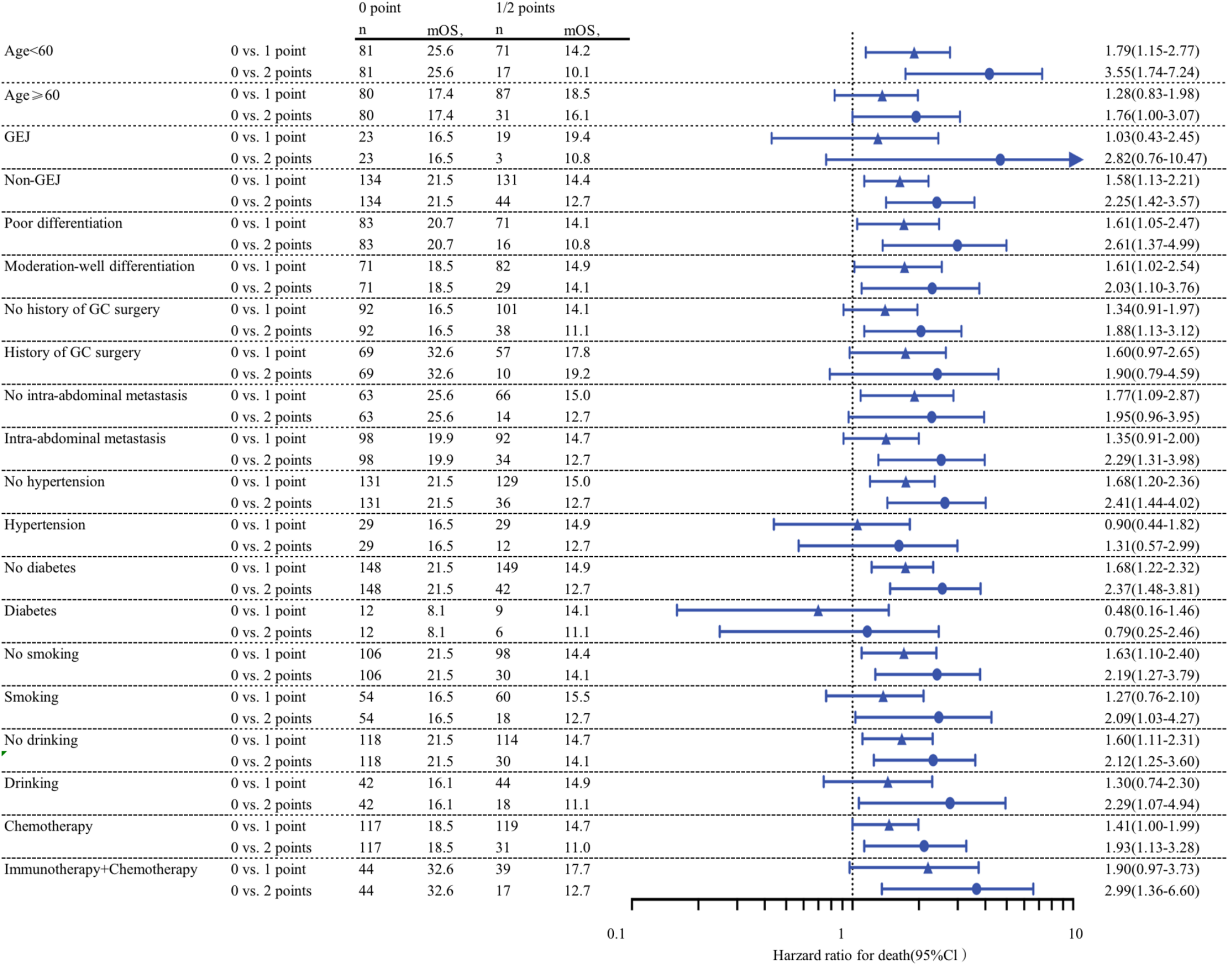
Hazard ratios and median OS comparing MPS categories (0 vs. 1 point and 0 vs. 2 points) in subgroups analysis. GC, gastric cancer; GEJ, gastroesophageal junction.

We further categorized the patients into two subgroups: the MPS‐low group (MPS 0) and the MPS‐high group (MPS 1 or 2). Figure 4 shows the forest plot for subgroups in all patients; significant differences in survival were observed in patients with age <65 and ≥65 years, non‐GEJ, poor and moderate‐well differentiation, no hypertension, no diabetes, no smoking, no drinking, and use of chemotherapy alone. Patients in the MPS‐low group experienced superior OS compared with the MPS‐high group across almost all subgroups.

## 4 DISCUSSION

The primary objective of this study was to assess the prognostic value of the MPS in advanced GC. Consistent with the findings in mPDAC reported by Lebenthal et al.,^20^ our study confirmed that the MPS serves as an independent prognostic factor for patients with advanced GC, the median OS was 20.7, 14.9, and 12.7 months for MPS 0, MPS 1, and MPS 2(*p* < 0.001). The 2‐year OS rates were 47.4%, 31.8%, and 24.1% for patients with MPS 0, 1, and 2, respectively. Importantly, our research included patients who underwent a combination of immunotherapy and chemotherapy, and the MPS demonstrated comparable predictive value within this subgroup. Remarkably, in subgroup analyses, patients who did not meet the criteria of an NLR of 4 or higher and albumin levels of 4 g/dL or lower (MPS = 0) exhibited an excellent prognosis, in contrast to patients who met either or both of these criteria.

There is a growing body of evidence indicating that the combination of preoperative albumin concentration and the NLR holds predictive value for DFS and OS in patients with Stage I–II GC.^22^, ^23^ Additionally, neutrophil count and albumin level 1 month after curative surgery reflect long‐term prognosis.^24^ Previous study has also shown a correlation between higher NLR and lower serum albumin levels and more advanced clinical staging in GC.^25^ These data suggested that the MPS may be effective in advanced GC. Our study contributes novel evidence supporting the potential utility of the combined NLR and serum albumin as a prognostic biomarker in advanced GC, shedding light on a previously unexplored aspect. Furthermore, our study suggested that the evaluated scoring system holds prognostic significance for patients receiving first‐line immunotherapy as well. Particularly, patients with MPS 0 exhibited significantly better prognosis in this subgroup. These findings indicated the promising potential of the MPS in the era of immunotherapy and highlighted its potential clinical utility.

The cut‐off value of the NLR utilized in our scoring system is strongly supported by previous studies, including a meta‐analysis involving more than 5000 individuals,^26^ as well as a large‐scale study conducted by Shimada et al.^27^ The albumin cut‐off value employed in our study corresponded to the lower limit of the normal range commonly used in clinical practice. Notably, the MPS is straightforward to calculate and relies solely on readily available clinical data. Furthermore, our choice of first‐line chemotherapy regimens aligns with the recommendations outlined in the current National Comprehensive Cancer Network (NCCN) guidelines for GC,^28^ enhancing the practicality of implementing this scoring system in both clinical and research settings. Given the demonstrated prognostic advantage of the MPS in mPDAC, it holds the potential to serve as a universal prognostic tool for various solid tumors, particularly in advanced stages.

The observed associations between the MPS and prognosis may be explained by the influence of heightened inflammation and compromised nutritional status. In this respect, in 1863, Virchow established that the production of inflammatory cytokines is an important step in tumor development and progression.^29^ Furthermore, these cytokines stimulate tumor‐induced immune suppression,^30^ permitting tumor cells to escape host immune resistance.^31^ Neutrophils have been recognized as an important component of the inflammatory infiltrate in the TME.^32^ Current evidence suggests neutrophils mediate angiogenesis through mitogenic and proangiogenic molecules, including elastase, prokineticin 2, and metallopeptidase 9 (MMP9).^33^ On the other hand, tumor‐associated neutrophil has been proven to be induced by the immunosuppressive cytokine transforming growth factor‐beta (TGF‐β) in the cancer setting to acquire a protumorigenic (N2) phenotype, playing a role in tumor growth, invasion, angiogenesis, and immunosuppression.^34^, ^35^ Lymphopenia may affect all or some T or B lymphocyte subpopulations,^36^ limiting the critical mechanisms of immune function against cancer.^37^ T lymphocytes represent the core cells that mediate anti‐tumor immunity and are the targets of immune checkpoint therapy.^38^ Various studies have shown that lymphopenia is correlated with shorter survival in different types of cancers.^39^, ^40^ In advanced cancers, serum albumin levels are frequently used as a laboratory parameter to indicate symptoms related to inflammatory response or cancer cachexia.^41^ There is a rich literature available substantiating that a low albumin level may be associated with poor prognosis in various cancers, including GC.^17^, ^42^, ^43^ The MPS, which combines an inflammatory marker and serum albumin, offers a comprehensive assessment of both nutritional status and inflammation, exhibiting a good performance as an independent prognostic index in mPDAC and in advanced GC. For individual patients, this scoring system can function as an initial screening tool and physicians can conveniently calculate the MPS value by extracting commonly used clinical indicators, empowering them to make more informed decisions regarding patient management promptly. While the AUC of the MPS may be comparatively low, it still possesses significant potential in clinical applications, serving as an additional supplement to the TNM staging in the clinical setting. Furthermore, the MPS deserves further investigation, as it provides a foundation for the development of more clinically valuable models.

However, the limitations of this research should be acknowledged. First of all, our findings were based on patients from a single center. Conducting a prospective multicenter study would enhance the robustness and applicability of the findings. Moreover, the study lacked pathological data and molecular characteristics of included patients, including major mismatch repair status, human epidermal growth factor receptor 2 status, and PD‐L1 expression, were insufficient. Despite the above mentioned limitations, this scoring system has huge prospects for clinical application but warrants further validation in larger populations and extension to other tumor types.

## 5 CONCLUSION

In the rapidly evolving landscape of tumor therapy, the need for a concise prognostic marker is paramount. Our findings indicate that the MPS represents a cost‐effective and straightforward clinical prognostic model for advanced GC. This prognostic scoring system can serve as a valuable reference for clinical decision‐making and holds promising prospects for application in medical practice. Nonetheless, it is crucial to emphasize that further studies with larger sample sizes are indispensable to validate the reliability and robustness of our findings.

## AUTHOR CONTRIBUTIONS

**Mingyu Wan:** Data curation (equal); formal analysis (equal); writing – original draft (equal). **Yongfeng Ding:** Data curation (equal); formal analysis (equal); writing – original draft (equal). **Xiaolu Ma:** Formal analysis (equal). **Xiaoyu Chen:** Investigation (equal). **Xin Xu:** Project administration (equal). **Chenyu Mao:** Project administration (equal). **Jiong Qian:** Supervision (equal). **Cheng Xiao:** Investigation (equal). **Haiping Jiang:** Validation (equal). **Yulong Zheng:** Validation (equal). **Lisong Teng:** Conceptualization (equal). **Nong Xu:** Conceptualization (equal); funding acquisition (equal); writing – review and editing (equal).

## FUNDING INFORMATION

This work was supported by grants from Natural Science Foundation of Zhejiang Province (LQ19H160031).

## CONFLICT OF INTEREST STATEMENT

The authors report no conflicts of interest in this work.

## ETHICS STATEMENT

The study was conducted in accordance with the Declaration of Helsinki, the protocol was approved by Ethics Committee of Zhejiang University (reference number: 2022–871). Individual consent was needless for this retrospective analysis.

## Supporting information


Figure S1.
Figure S2.
Figure S3.
Figure S4.

## Data Availability

Data supported the findings of the study could be acquired from the corresponding author by reasonable request.
