# Supplementary material for: The Memorial Sloan Kettering Prognostic Score: Correlation with survival in patients with advanced gastric cancer
Source: Cancer Med. 2023 Oct 3;12(19):19656–66. doi: 10.1002/cam4.6608 (PMC10587931; doi:10.1002/cam4.6608)
Supplement: Supplementary file 1 — Figure S1. Figure S2. Figure S3. Figure S4. [file CAM4-12-19656-s001.docx]

**Supplementary material**

**
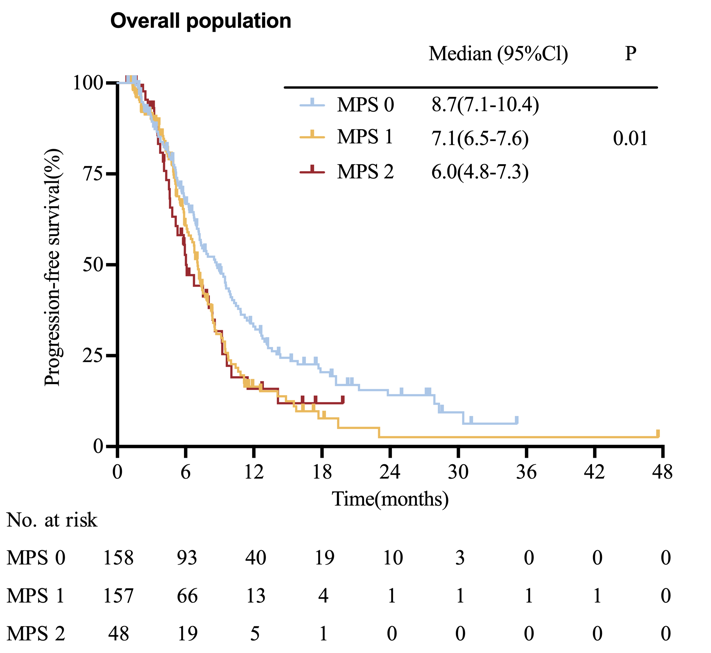
**

**Supplemental Figure 1.** Kaplan-Meier curves for PFS in all patients. *PFS*, progression-free survival, *MPS* Memorial Sloan Kettering Prognostic Score.

**
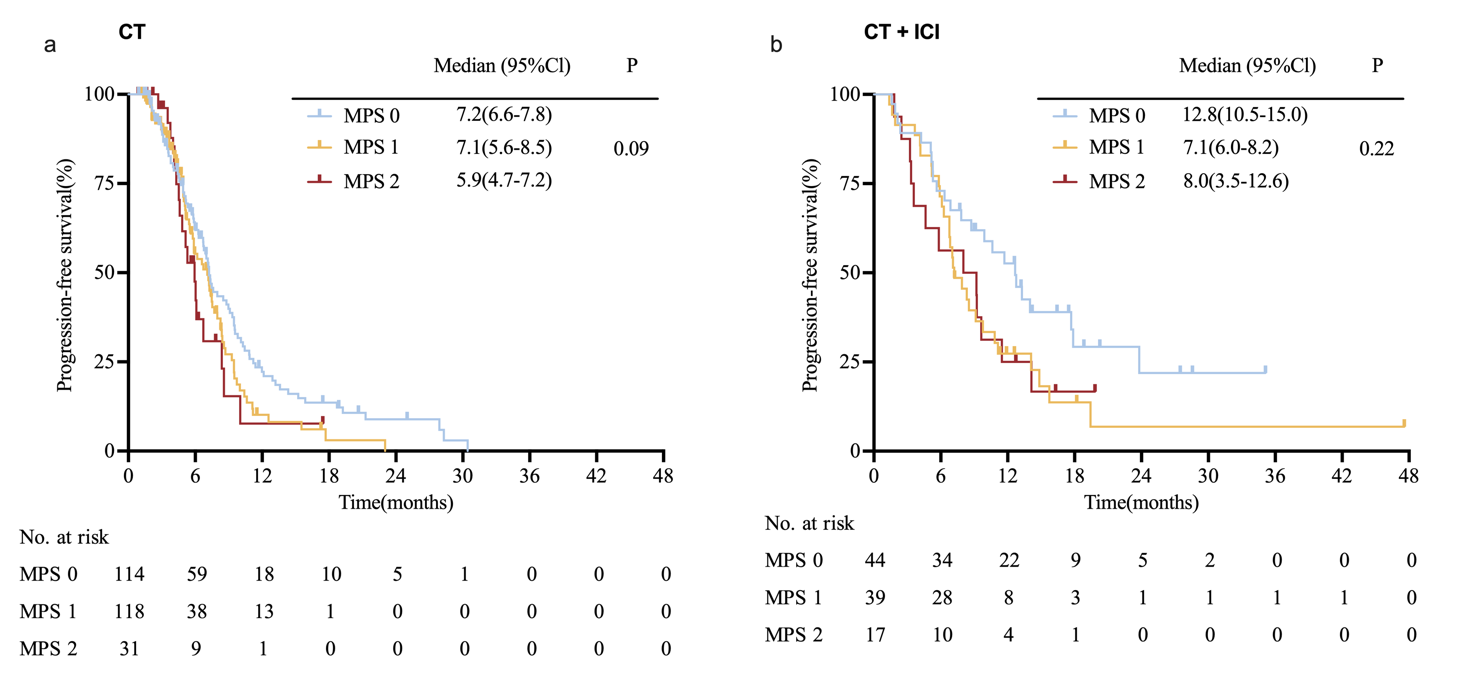
**

**Supplemental Figure 2.** PFS analysis according to the MPS in patients with advanced GC. (a) Kaplan–Meier curves for PFS in patients who received chemotherapy alone; (b) Kaplan–Meier curves for PFS in patients who received immunotherapy plus chemotherapy. *PFS*, progression-free survival, *MPS* Memorial Sloan Kettering Prognostic Score.


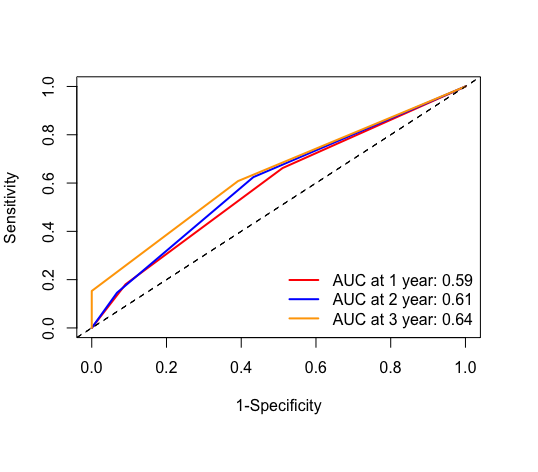


**Supplemental Figure 3.** Receiver operating characteristic (ROC) curves for the MPS for predicting 1-year, 2-year and 3-year OS.

**
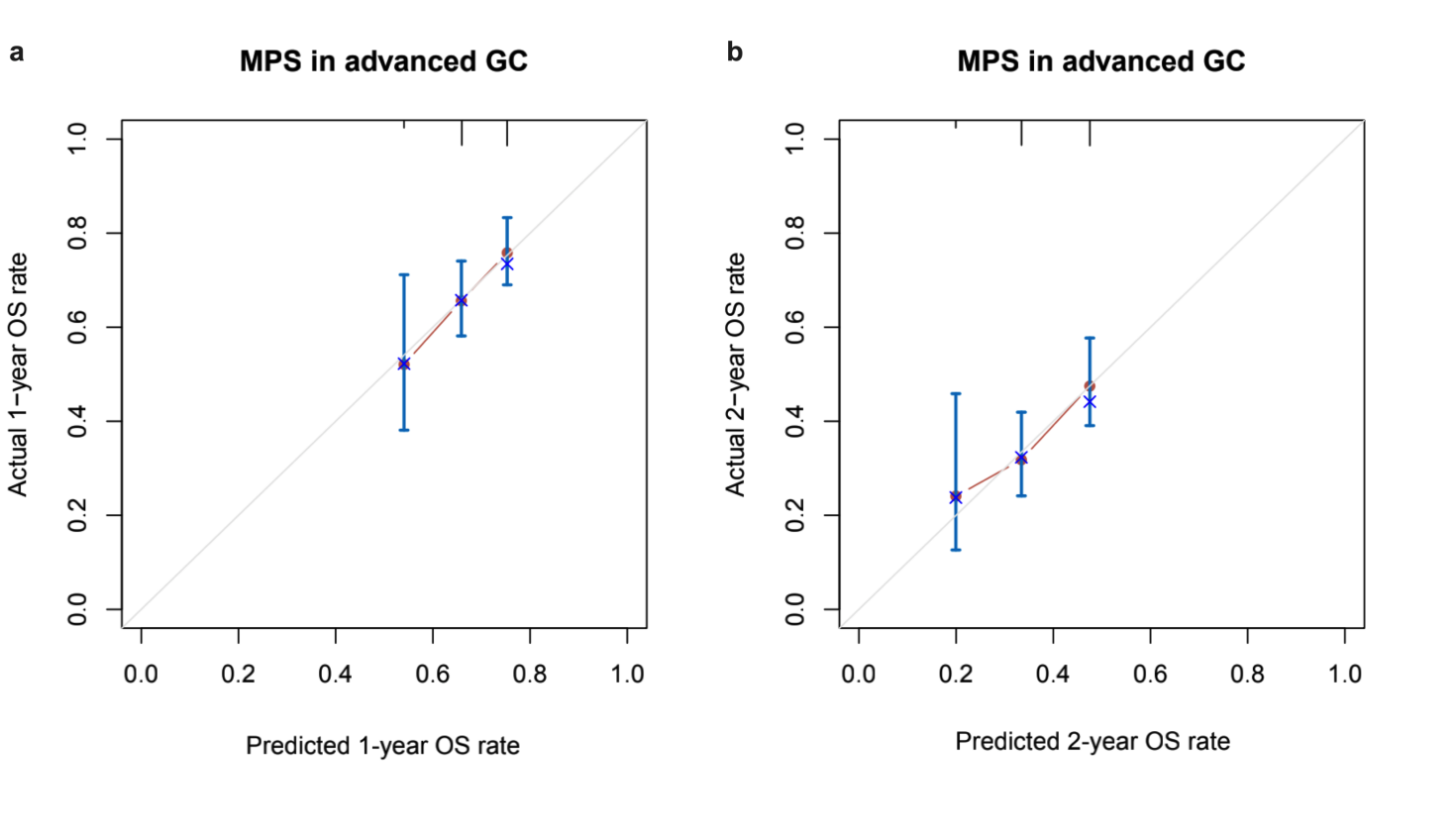
**

**Supplemental Figure 4.** Calibration plots for the MPS to predict the probability of 1-year and 2-year survival of advanced gastric cancer. (a) calibration plots for 1‐year survival; (b) calibration plots for 2‐year survival.
